# Cold chain and virus‐free chloroplast‐made booster vaccine to confer immunity against different poliovirus serotypes

**DOI:** 10.1111/pbi.12575

**Published:** 2016-06-01

**Authors:** Hui‐Ting Chan, Yuhong Xiao, William C. Weldon, Steven M. Oberste, Konstantin Chumakov, Henry Daniell

**Affiliations:** ^1^ Department of Biochemistry School of Dental Medicine University of Pennsylvania Philadelphia PA USA; ^2^ Centers for Disease Control and Prevention Atlanta GA USA; ^3^ Center for Biologics Evaluation and Research Food and Drug Administration Bethesda MD USA

**Keywords:** human infectious diseases, molecular farming, chloroplast transformation, bioencapsulation, oral delivery, mucosal immunity

## Abstract

The WHO recommends complete withdrawal of oral polio vaccine (OPV) type 2 by April 2016 globally and replacing with at least one dose of inactivated poliovirus vaccine (IPV). However, high‐cost, limited supply of IPV, persistent circulating vaccine‐derived polioviruses transmission and need for subsequent boosters remain unresolved. To meet this critical need, a novel strategy of a low‐cost cold chain‐free plant‐made viral protein 1 (VP1) subunit oral booster vaccine after single IPV dose is reported. Codon optimization of the VP1 gene enhanced expression by 50‐fold in chloroplasts. Oral boosting of VP1 expressed in plant cells with plant‐derived adjuvants after single priming with IPV significantly increased VP1‐IgG1 and VP1‐IgA titres when compared to lower IgG1 or negligible IgA titres with IPV injections. IgA plays a pivotal role in polio eradication because of its transmission through contaminated water or sewer systems. Neutralizing antibody titres (~3.17–10.17 log_2_ titre) and seropositivity (70–90%) against all three poliovirus Sabin serotypes were observed with two doses of IPV and plant‐cell oral boosters but single dose of IPV resulted in poor neutralization. Lyophilized plant cells expressing VP1 stored at ambient temperature maintained efficacy and preserved antigen folding/assembly indefinitely, thereby eliminating cold chain currently required for all vaccines. Replacement of OPV with this booster vaccine and the next steps in clinical translation of FDA‐approved antigens and adjuvants are discussed.

## Introduction

Despite their success, live attenuated oral poliovirus vaccine (OPV) and inactivated poliovirus vaccine (IPV) have a number of shortcomings making them less than optimal for their use in posteradication environment (Mueller *et al*., 2005; Racaniello, 2011). Sabin strains used in OPV can revert to virulence by point mutations or recombination with other enteroviruses (Burns *et al*., 2014; Runckel *et al*., 2013), while IPV is expensive, does not induce adequate mucosal immunity (Onorato *et al*., 1991), requires cold storage/transportation and intramuscular injections. Genetic instability of Sabin strains was observed several decades ago among cases of vaccine‐associated paralytic poliomyelitis in recipients of OPV in the USA and their close contacts (Alexander *et al*., 2004; Schonberger *et al*., 1976). Outbreak in Haiti and Dominican Republic (Kew *et al*., 2002) a decade ago led to the discovery of circulating vaccine‐derived polioviruses (VDPV) that caused numerous outbreaks of all three serotypes (Diop *et al*., 2015; Lakhani and Bumb, 2014). Immunocompromised patients can be chronically infected with poliovirus following vaccination with OPV (Sutter and Prevots, 1994) and continue to excrete virulent poliovirus for years and even decades (Dunn *et al*., 2015). The inevitability with which virulent VDPV emerge led WHO to propose complete withdrawal of OPV after the circulation of wild polioviruses has been stopped, and to replace it with the global use of IPV to prevent re‐emergence of poliovirus by maintaining adequate population immunity (Bhasin, 2008).

This posteradication scenario is complicated by several factors linked to the properties of IPV. Even though persons immunized with IPV are completely protected against paralysis, they could be infected with poliovirus and transmit it to their contacts. Additionally, poliovirus can live in an infected person's faeces for many weeks and can also contaminate food and water in unsanitary conditions. Therefore, virulent polioviruses can silently circulate in communities immunized with IPV (Shulman *et al*., 2014). While mucosal immunity caused by OPV is significantly more effective, it rapidly wanes, but can be boosted by either OPV or IPV (Duintjer Tebbens *et al*., 2013). The cost and limited supply of IPV has led Strategic Advisory Group of Experts (SAGE) to propose that only one dose of IPV can be used in some countries to prime population immunity, even though one dose of IPV does result in complete seroconversion. This suggests that there is a need for booster vaccine that could be used in previously immunized individuals to induce anamnestic response and stimulate mucosal immunity. Poliovirus is an intestinal virus transmitted from person to person through the mouth and it multiplies in the intestine. The intestinal mucosal surface plays a pivotal role during poliovirus infection. Thus, building up a localized pathogen‐specific mucosal immune response is important as the antibodies generated can neutralize the virus before it can cause infection (Shakya *et al*., 2016).

Plant‐derived subunit vaccines are heat‐stable and are free from contamination with animal pathogens. They can also be engineered to contain multiple antigens and transmucosal carriers, to protect against multiple infectious diseases (Albarracín *et al*., 2015; Chan and Daniell, 2015). Upon oral delivery, protein drugs expressed in plant cells are protected from acids and enzymes in the stomach by the plant cell wall; when fused with the transmucosal carrier protein drugs released in the gut lumen are efficiently delivered to the circulatory or immune system (Arlen *et al*., 2008; Belyakov and Ahlers, 2009; Davoodi‐Semiromi *et al*., 2010; Kohli *et al*., 2014; Shenoy *et al*., 2014; Sherman *et al*., 2014; Shil *et al*., 2014; Su *et al*., 2015). Such mechanistic and conceptual advances could revolutionize vaccine delivery by eliminating the cost of complex production systems, such as fermentation, purification, cold storage and transportation (Jin and Daniell, 2015; Kwon *et al*., 2013). Although potato‐derived HBsAg expressed via the nuclear genome was tested in preclinical and in human clinical trials a decade ago (Kong *et al*., 2001; Thanavala *et al*., 2005), progress in advancing to later stages is slow. Two major challenges are the low levels of expression of antigens via the nuclear genome and the potential to induce tolerance without injectable priming of antigens with adjuvants (Chan and Daniell, 2015; Rybicki, 2014). Both these concerns are addressed in chloroplast‐derived vaccine antigens (Chan and Daniell, 2015; Lössl and Waheed, 2011).

In order to address inadequacies of the current polio vaccines, including insufficient vaccine efficacy in some populations, instability and reversion to neurovirulence, shedding of circulating VDPV, and the high cost and inadequate mucosal immunity of IPV, a low‐cost booster vaccine has been developed in this study using polio viral antigen expressed in chloroplasts. The strategy of using a plant‐made VP1 subunit vaccine for an oral booster rather than repeated OPV vaccination is a novel approach to achieve the goal of global poliovirus (PV) eradication. In this study, we provide evidence that oral boosting with chloroplast‐derived VP1 together with plant‐made adjuvants (saponin and squalene) induces strong immune responses that confer protective immunity against different PV serotypes.

## Results

### VP1 chloroplast vectors

**Figure 1:**
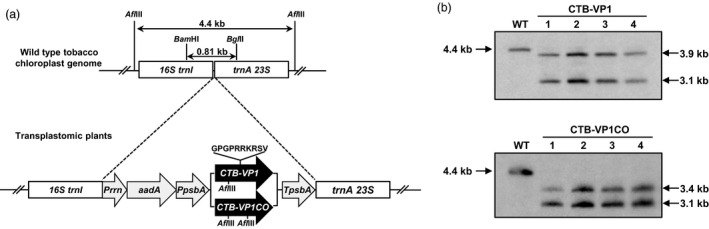
Creation and characterization of transplastomic tobacco lines expressing native and codon‐optimized CTB‐VP1. (a) tobacco chloroplast transformation vectors containing CTB‐VP1 expression cassettes. *Prrn*, rRNA operon promoter; *aadA*, aminoglycoside 3'‐adenylyltransferase gene; *PpsbA*, promoter and 5'‐UTR of the *psbA* gene; *CTB*, coding sequence of nontoxic cholera B subunit; *VP1*, coding sequence for poliovirus VP1 gene; *TpsbA*, 3'‐UTR of the psbA gene; *trnI*, isoleucyl‐tRNA;* trnA*, alanyl‐tRNA; (b) Southern blot analysis of native and codon‐optimized CTB‐VP1 transplastomic tobacco lines. *Afl* III‐digested wild type (WT) and transformed (line 1, 2, 3 and 4) genomic DNA was probed with DIG‐labelled flanking sequence digested with *Bam* HI/*Bgl*II. UTR, untranslated region.

Poliovirus capsid viral protein 1 (VP1) is one of the poliovirus structural proteins and could serve as an ideal vaccine candidate. Therefore, two VP1 proteins (native and codon optimized) derived from Sabin 1 coding sequences fused with the transmucosal carrier CTB were expressed in tobacco and lettuce chloroplasts. Of the 302 amino acids in this protein, 187 codons were optimized by changing the codon usage frequency to resemble that of the chloroplast *psbA* gene (the most highly translated chloroplast gene). Rare codons were replaced with optimal codons for transgene expression in chloroplasts and the AT content of the optimized VP1 gene increased from 51.98% to 59.03%. Both CTB‐VP1 fusion genes were constructed with a GPGP (Gly‐Pro‐Gly‐Pro) hinge region to minimize steric hindrance of the fused VP1, as well as a furin cleavage site, RRKRSV (Arg‐Arg‐Lys‐Arg‐Ser‐Val) (Figure 1a). Both fusion genes were driven by the *psbA* promoter and 5'‐untranslated region (UTR) to increase expression, and the transcript was stabilized by the *psbA* 3'‐UTR.

### Integration of VP1 into tobacco plastomes

CTB‐VP1 transplastomic tobacco lines were confirmed by PCR analysis with primer sets 3P/3M and 5P/2M. Targeted integration and homoplasmy of the CTB‐VP1 gene were further evaluated by Southern blot probed with the *trnI* and *trnA* flanking sequence. All independent transplastomic tobacco lines showed distinct hybridization fragments with the correct size, but not the 4.4‐kb fragment from wild type in the *Afl*III‐digested total DNA blot (Figure 1a,b). Thus, Southern blot analysis confirmed the site‐specific stable integration of the transgenes into the chloroplast genome and homoplasmy.

### Folding, stability and CTB‐VP1 pentamer assembly in lyophilized tobacco leaves

**Figure 2:**
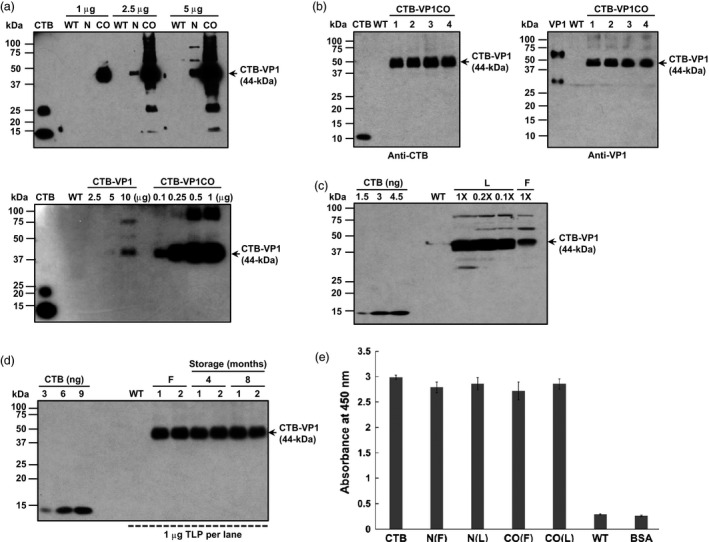
Characterization of CTB‐VP1 transplastomic lines. (a) Western blot of CTB‐VP1 native (N) or codon optimized (CO) and untransformed plant extracts loaded at indicated total protein (top) or serial dilution (bottom) and probed with anti‐CTB antibody. CTB (5 ng) was loaded as a positive control. (b) Western blot of CTB‐VP1 in four independent transplastomic lines and wild type (WT) (1 μg/lane) controls probed with anti‐CTB or anti‐VP1 and protein standards. Antibodies used (dilution factor): rabbit anti‐CTB polyclonal antibody 1 : 10 000 dilution; rabbit anti‐VP1 polyclonal antibody 1 : 1000 dilution. (c) Comparison of CTB‐VP1 in lyophilized (L) or frozen (F) leaf samples; 10 mg ground powder was extracted in 300 μL of extraction buffer. 1× is 1 μL of ground extract. (d) CTB‐VP1 stability after storage of lyophilized leaves at ambient temperature for 4 or 8 months (e) GM1 binding assay of native (N) and codon‐optimized CTB‐VP1 (CO). F: fresh; L: lyophilized; WT: untransformed; BSA (1%, w/v), bovine serum albumin. Data are means ± SD of three independent experiments.

CTB‐VP1 accumulation in transplastomic plants was quantified by Western blot analysis. Intensities of CTB‐VP1 polypeptides in native and codon‐optimized plants were compared with known amounts of CTB standard. The codon‐optimized VP1 sequence (2600 μg/g dry weight) increased expression by 50‐fold when compared with the native VP1 gene product (54 μg/g DW, Figure 2a). Native and codon‐optimized CTB‐VP1 reached up to 0.1% and 4–5% of the total leaf protein, respectively. As shown in Figure 2b, the monomer CTB‐VP1 fusion protein with the correct molecular mass of 44 kDa was detected with anti‐CTB or VP1 antibody. CTB‐VP1 antigen increased further ~20‐fold in lyophilized cells when compared with frozen leaf samples (Figure 2c). The intact monomer band of CTB‐VP1 fusion proteins was observed without any detectable degradation of CTB‐VP1 in all tested lyophilized samples after storage for 4 and 8 months at ambient temperature (Figure 2d). Formation of pentameric structures of the CTB‐VP1 expressed in chloroplasts was evaluated using GM1 binding ELISAs. As shown in Figure 2e, both native and codon‐optimized fresh and lyophilized CTB‐VP1 from tobacco showed comparable absorbance to CTB (positive control). This indicates that CTB‐VP1 fusion protein expressed in both fresh and lyophilized chloroplasts formed proper pentameric structures that could bind the GM1‐ganglioside receptor, which is a requirement for oral protein drug delivery. The stability of VP1, efficacy of binding to GM1‐ganglioside receptor, proper folding and pentamer assembly were maintained after lyophilization and prolonged storage for 8 months at ambient temperature (Figure 2d,e).

### Evaluation of antibody responses to VP1

**Figure 3:**
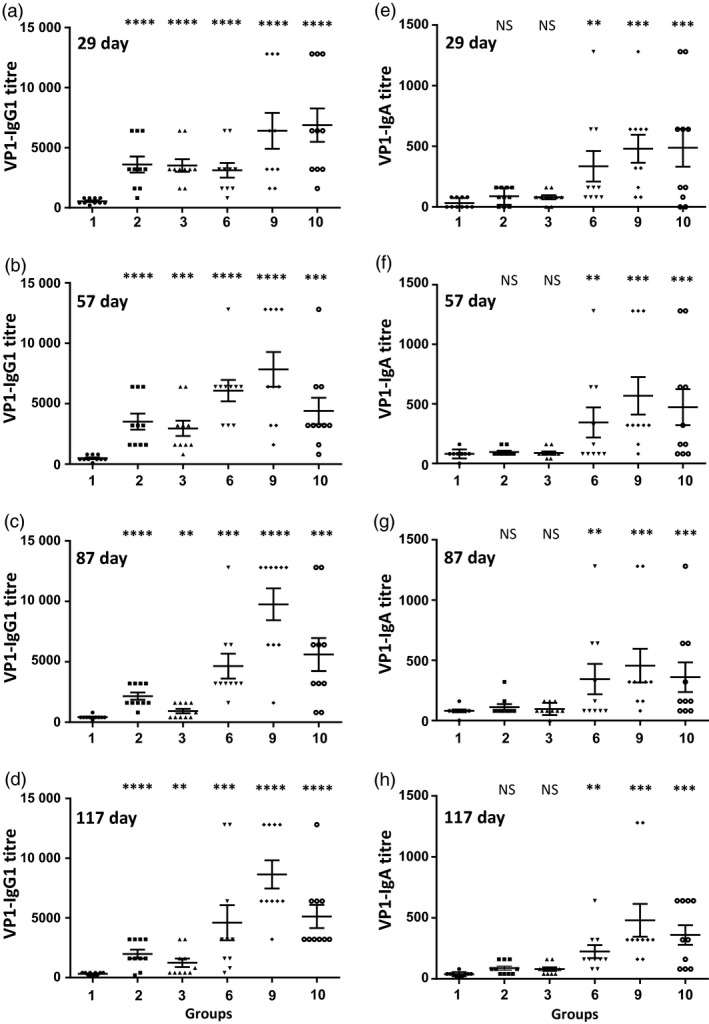
Evaluation of serum VP1‐IgG1 and VP1‐IgA antibody titres after oral or subcutaneous vaccination. Antibody responses of mice (*n *=* *10/group) vaccinated with single or two doses of inactivated poliovirus vaccine (IPV) or VP1 bioencapsulated in plant cells. (a–d) VP1‐IgG1 antibody titres at different time points: (a, b) weekly boosts and sera samples collected on days 29 and 57; (c, d) monthly boosts and samples collected on days 87 and 117; (e–h) VP1‐IgA antibody titres at different time points: (e, f) weekly boosts and sera samples collected on days 29 and 57; (g, h) monthly boosts with sera samples collected on days 87 and 117. Group 1: untreated; group 2: 2 doses of IPV; group 3: IPV single dose; group 6: IPV prime, boosted with native VP1 or group 9: codon‐optimized VP1 in plant cells with adjuvants (saponin/squalene); group 10: same as group 9 but without IPV priming. Results are shown as individual reciprocal endpoint antibody titres and mean ± SEM. One‐way ANOVA showed significant differences between groups (*P* < 0.0001) and *post hoc* comparisons by *t*‐test showed significant differences between specific treatment groups and respective control group. (**P <* *0.05, ***P <* *0.01, ****P <* *0.001, *NS* not significant).

**Figure 4:**
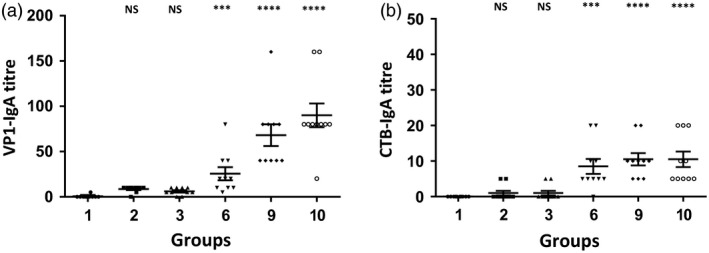
Faecal IgA antibody titres after oral and subcutaneous vaccination. Levels of VP1 and CTB‐specific IgA antibody titres in faecal extracts obtained from mice (*n* = 10/group) after 3‐month vaccination. Faecal pellets were collected at day 10 after oral boosting. ELISA titres are shown (a) VP1‐specific IgA antibody titre. (b) CTB‐specific IgA antibody titre. Group 1: untreated; group 2: 2 doses of inactivated poliovirus vaccine (IPV); group 3: IPV single dose; group 6: IPV prime, boosted with native VP1 or group 9: codon‐optimized VP1 in plant cells with adjuvants (saponin/squalene); group 10: same as group 9 but without IPV priming. Antibody titres are defined as the reciprocal of the highest dilution above the cut‐off, which was three times the mean background. All samples were tested in triplicate. Results are shown as individual reciprocal endpoint antibody titres and mean ± SEM. One‐way ANOVA showed significant differences between groups (*P* < 0.0001) and *post hoc* comparisons by *t*‐test showed significant differences between specific treatment groups and the control group. (***P <* *0.01, ****P <* *0.001, *NS* not significant).

**Figure 5:**
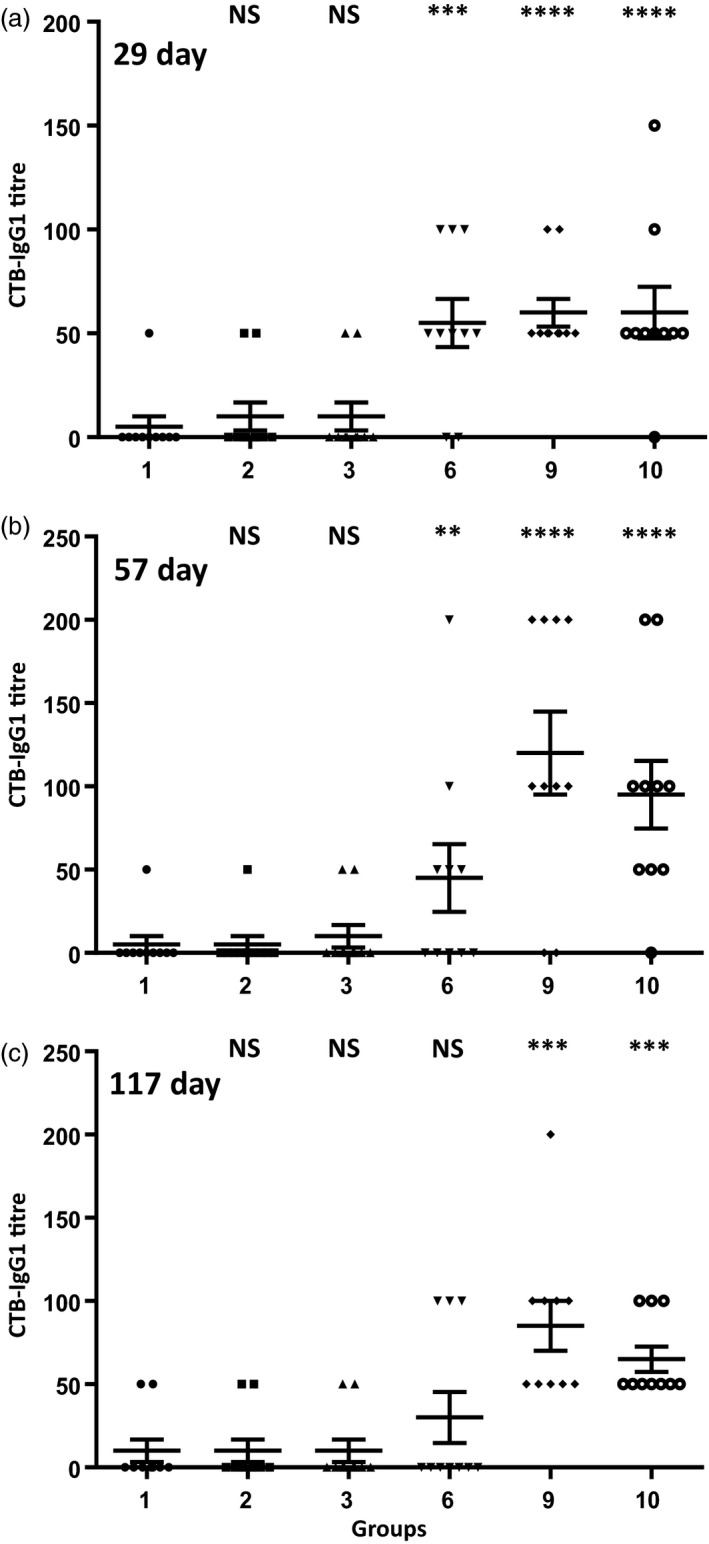
Evaluation of serum CTB‐IgG1 antibody titres after oral vaccination. Cholera toxin B subunit was coated on ELISA plates and probed with individual heat‐inactivated sera samples (starting with a 1 : 50 dilution). CTB‐IgG1 antibody titres at different time points: (a, b) weekly boosts and sera samples collected on days 29 and 57; (c) monthly boosts and samples collected on day 117. Group 1: untreated; group 2: 2 doses of inactivated poliovirus vaccine (IPV); group 3: IPV single dose. group 6: IPV prime, boosted with native VP1 protein with adjuvants; group 9: IPV prime, boosted with codon‐optimized VP1 protein with adjuvants; group 10: boosted with codon‐optimized VP1 with adjuvants but without IPV priming. Saponin and squalene were used as adjuvants. Results are shown as individual reciprocal endpoint antibody titre and mean ± SEM. One‐way ANOVA showed significant differences between groups (*P* < 0.0001) and *post hoc* comparisons by *t*‐test showed significant differences between specific treatment groups and the control group. (**P <* *0.05, ***P <* *0.01, ****P <* *0.001, *NS* not significant).

The objective of this study was to determine whether lyophilized CTB‐VP1 protein formulated with plant‐derived adjuvants (saponin and/or squalene) approved by FDA could induce specific antibody immunogenicity and neutralize different poliovirus serotypes. We investigated the impact of adjuvants (squalene and saponin) using plant cells expressing low level of VP1 (native gene) as we anticipated that higher antigen dose might mask the adjuvant effect. Most importantly, we could use similar weight of plant cells but with ~50‐fold lower antigen dosage, thereby eliminating potential impact of naturally present adjuvants in plant cells. Mice boosted with native (1.08 μg VP1 per dose, group 6) or codon‐optimized tobacco‐derived CTB‐VP1 (52 μg VP1 per dose, group 9) with both adjuvants had anti‐VP1 IgG1 mean antibody titres of 6080 or 7840 on 57th, 4640 or 9760 87th 4640 or 8640 117th days. In contrast, two doses of IPV (group 2) resulted in 3520, 2160 or 1980 VP1 titres when compared to a single dose (group 3) of IPV (2960, 920 or 1240, Figure 3b–d). Therefore, VP1‐IgG1 titres reached highest levels in the first month and did not increase after subsequent oral boosters (Figure 3a–d). However, VP1‐IgG1 titres in IPV single or two doses declined after the first month. Similarly, serum VP1‐IgA titres increased after oral boosting in the first month (group 6: 336, 344, 264 or 224; group 9: 480, 552, 400, 480) and did not increase significantly after subsequent boosting (Figure 3e–h). In sharp contrast, IPV single or double doses did not increase IgA titres, confirming limitation of systemic vaccine delivery. To investigate mucosal immune response, we measured VP1 specific from faecal extracts (Figure 4a). The highest VP1‐IgA titres were showed in groups of mice (groups 6, 9 and 10) orally boosted with native or codon‐optimized VP1 formulation. These results show that oral boosting with plant cells expressing CTB‐VP1 can induce both mucosal and systemic immune responses whereas IPV single or double dose developed lower levels of IgG1 and negligible IgA titres. Because both the native and codon‐optimized VP1 are fused with CTB, we also measured specific CTB‐IgG1 antibody titres in serum and faecal extracts. Most of the groups showed negligible titres (50–150) in serum (Figure 5) and faecal extracts (Figure 4b), well below the standard deviation of VP1‐IgG1 titres and are therefore considered negligible.

### Poliovirus‐neutralizing titres against all Sabin 1, 2 and 3 strains following priming and boosting

**Figure 6:**
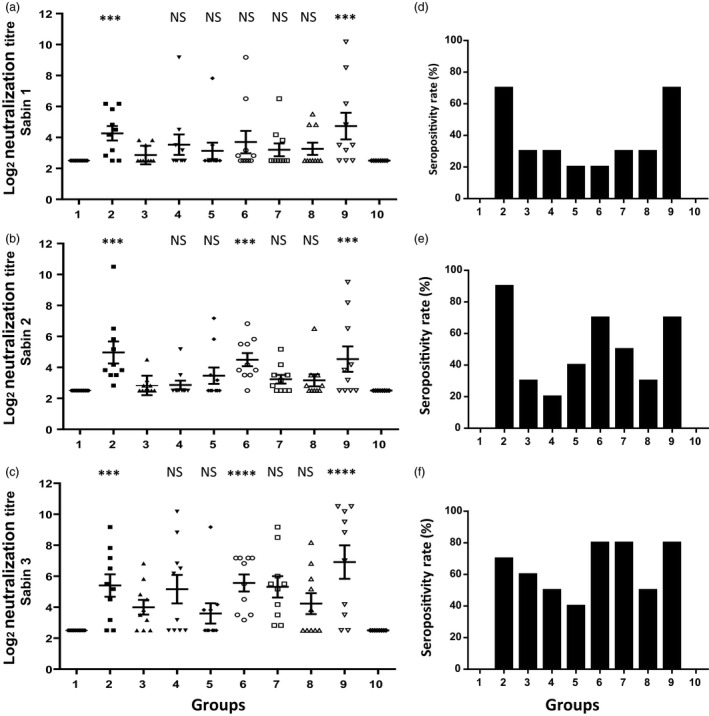
Determination of poliovirus‐neutralizing titres and seropositivity rate of Sabin 1‐, 2‐ and 3‐neutralizing titres after subcutaneous inactivated poliovirus vaccine (IPV) or oral VP1 boosting. Virus‐neutralizing antibody titres of 117 days sera from mice (*n* = 10/group) boosted with native or codon‐optimized CTB‐VP1 antigens adjuvanted with saponin only (groups 4 and 7), squalene only (groups 5 and 8) or both (groups 6, 9 and 10); mice with two doses of IPV (group 2) or single IPV dose (group 3); and untreated mice (group 1). Individual titres for each mouse were plotted, and the bar represents the mean neutralizing titre ± SEM. The serum dilution of a reciprocal titre at which no virus neutralization was detected was recorded as the log_2_ (titre) of 2.5. Poliovirus‐neutralizing antibodies against all three Sabin strains, (a) Sabin 1, (b) Sabin 2 and (c) Sabin 3. One‐way ANOVA showed significant differences between groups (*P* < 0.0001) and *post hoc* comparisons by *t*‐test showed significant differences between specific treatment groups and group 3 – single IPV dose (***P <* *0.01, ****P <* *0.001, *NS* not significant). The seropositivity rate of poliovirus‐neutralizing antibodies as determined by the number of mice with seroprevalence (neutralizing antibody log_2_ (titre) ≥3) with the total number of mice in each group boosted with the native or codon‐optimized CTB‐VP1 (groups 4–10), or, IPV two doses (group 2) at day 1 and day 30 or IPV single dose (group 3). The seropositivity rate of neutralizing titres against Sabin strains 1, 2 and 3 (d–f) are shown.

Blood samples from all experimental and untreated groups were tested in a double‐blind manner and in triplicate samples at CDC. A serum sample was considered seropositive if antibodies were present at a log_2_ titre ≥2.5. Individual neutralization titres were plotted, and the bar represents the mean neutralizing titre ± SEM of each group. Results show that after IPV priming, all experimental groups – oral boosting with the native (groups 4–6) or codon‐optimized VP1 antigen with plant‐derived adjuvants (groups 7–9), as well as two doses of IPV (group 2) or single IPV dose (group 3) – induced different levels of neutralizing titres against all three Sabin strain serotypes. Results show that oral boosting with codon‐optimized VP1 (group 9) induced significantly higher Sabin 1‐, Sabin 2‐ and Sabin 3‐neutralizing antibodies (range ~3.17–10.17, ~3.17–9.5 and ~3.5–10.5 log_2_ titres), similar to the group of mice that received two doses of IPV (group 2) (range ~3.17–6.17, ~3.5–10.5 and ~3.17–9.17 log_2_ titres) (Figure 6a–c), but were significantly higher than those in mice with a single dose of IPV only (group 3) (range ~3.83, ~3.17–4.5 and ~2.5–6.8). However, no neutralizing antibodies were detected in sera from mice that were only orally boosted without IPV priming (group 10). Mice boosted with the native VP1 (group 6) had significantly lower Sabin 1‐neutralizing antibodies titres (range ~3.17–9.17) than mice boosted with codon‐optimized VP1 antigen (group 9) (~3.17–10.17) (*P *<* *0.01). However, mice boosted with native VP1 antigen formulation (group 6) showed significantly higher Sabin 2 and Sabin 3‐neutralizing antibodies titres than mice that received a single dose of IPV (group 3): (~3.5–6.83 and ~3.17–10.17 vs ~3.17–4.5 and ~2.5–6.8). Clearly, poliovirus neutralization studies show that only groups 2, 6 and 9 offer optimal protection against all three poliovirus serotypes. With the exception of group 6 in Sabin serotype 1, all three groups against all three serotypes provide statistically similar results.

To determine the seropositivity rate of poliovirus‐neutralizing antibodies, for each Sabin strain, the number of mice with seroprevalence (neutralizing antibody log_2_ (titre) ≥3) was compared with the total number of mice in each group. Mice receiving two doses of IPV (group 2) or orally boosted with codon‐optimized VP1 antigen with saponin and squalene adjuvants (group 9) showed highest seropositivity for poliovirus Sabin 1‐, 2‐ and 3‐neutralizing antibodies (Figure 6d,f). Seropositivity rate varied between 70% and 90% for two doses of IPV versus oral boosting with VP1. These results show that codon‐optimized VP1 antigen with plant‐derived adjuvants has the greatest seropositivity rate, neutralizing antibodies (Figure 6d–f) and virus‐neutralizing titres (log_2_ titre ~3.17–10.17) against all Sabin 1, 2 and 3 strains (Figure 6a–c).

## Discussion

September 2015 declaration of global eradication of type 2 poliovirus has led WHO SAGE to recommend the withdrawal of OPV2 from routine immunization programs in all countries, with concurrent introduction of at least one dose of IPV (the global polio eradication initiative (GPEI), 2015). However, multiple risks still remain in preparation for the global introduction of IPV and the upcoming switch from trivalent OPV to bivalent OPV, including limited IPV supply, persistent cVDPV transmission and challenges of poliovirus containment (GPEI Polio Eradication & Endgame Midterm Review, 2015). Most importantly, there is no booster technology available except IPV which is not affordable for many resource‐limited countries. After type 1 and type 3 polioviruses are eradicated, the routine use of OPV vaccination will be discontinued and replaced with global use of IPV. This transition is essential for maintaining high levels of population immunity to protect against the emergence of VDPV and future outbreaks. This underscores the need for development of a new generation of low‐cost polio vaccines because the current cost of IPV is too high for most developing countries (Verdijk *et al*., 2014). Although one should be cautious in extrapolation of results from rodent to human clinical applications, poor seroprotectivity and poliovirus neutralization obtained with a single dose of IPV reported in this study offer caution on the use of IPV without further boosting.

### Cold chain‐free polio booster vaccine

Expression of VP1 in chloroplasts and bioencapsulation in plant cells can protect antigens from the digestive system upon oral delivery and facilitates their release into the immune system in the gut by commensal microbes (Davoodi‐Semiromi *et al*., 2010; Limaye *et al*., 2006). CTB‐antigen fusions facilitate transmucosal delivery to the immune system via the GM1 intestinal epithelial receptor (Verma *et al*., 2010). Production of green vaccines against infectious diseases with ease of oral administration that does not require a cold chain is an important need, especially in areas with limited access to cold storage and transportation (Davoodi‐Semiromi *et al*., 2010). VP1 is also highly stable in lyophilized plant cells when stored at ambient temperature for several months, facilitating a cold chain‐free vaccine delivery system. Most importantly, lyophilization of plant cells maintains proper folding of CTB with disulphide bonds and pentamer assembly after prolonged storage at room temperature.

### Number of required oral boosters and formulations

Antigen‐specific IgG1 and IgA were significantly induced after few oral boosters which generated high levels of systemic and mucosal immunity. Both VP1‐IgG1 and VP1‐IgA titres reached highest levels after the first month of oral boosting and did not increase further with more boosters. Although neutralization data from later stage sera collection are provided here, previous batches evaluated for Sabin serotype 1 neutralization showed similar results in groups boosted with plant cells expressing VP1. Therefore, only one or two boosters of VP1 bioencapsulated in plant cells may be required. However, multiple boosters are now used for several vaccines. Current anthrax vaccine requires eight boosters (Lynch *et al*., 2014). Several doses of oral polio vaccine (as many as fifteen) were administered in outbreak areas in India (Lakhani and Bumb, 2014), but unfortunately, there were several cases of vaccine induced polio (Bhasin, 2008). To determine minimal number of boosts further experiments are required, although low cost and easy storage make the number of boosts less important than current vaccines that require cold storage and transportation. In this study, plant cells were suspended in phosphate‐buffered saline (PBS) before oral delivery but for delivery to children, suitable formulation with sugar syrup may be required. In this context, it should be added that oral delivery of lyophilized carrot cells expressing glucocerebrosidase, suspended in orange juice entered into Phase I/II clinical studies conducted by Protalix BioTherapeutics (Shaaltiel *et al*., 2015). Future studies should focus on expression of VP1 antigen in an edible plant suitable for oral delivery; several vaccine antigens and biopharmaceuticals have been expressed in lettuce chloroplasts (Daniell *et al*., 2016a,b; Jin and Daniell, 2015; Kanagaraj *et al*., 2011; Lakshmi *et al*., 2013; Ruhlman *et al*., 2007, 2010).

Although IPV is highly effective in inducing systemic antibodies to protect against paralytic disease, it is less efficient in inducing the mucosal immunity that is needed to prevent re‐infection and excretion of polioviruses into the environment especially in developing countries. Our results confirmed that in mice with single or two doses of IPV induced only minimal IgA titres, explaining the inadequate mucosal immunity of IPV. Mice primed with IPV and orally boosted with bioencapsulated VP1 elicited stronger antigen‐specific serum IgG1 and IgA titres in blood and faecal extracts, confirming that oral delivery of VP1 antigen with plant‐derived adjuvants generated both systemic and mucosal immune responses. Further, poliovirus infects the throat and intestines after contact with an infected person. As poliovirus invades via mucosal surfaces, localized pathogen‐specific mucosal immune responses are truly important because pathogen‐specific antibodies can neutralize pathogens before they can cause infection (Shakya *et al*., 2016). Thus, instead of repeatedly boosting with IPV, oral boosting with subunit VP1 formulation has the potential for development of a practical and efficient subunit poliovirus vaccine.

### Impact of antigen dosage

In this study, we evaluated both native and codon‐optimized VP1 antigens expressed in chloroplasts. The level of VP1 protein was 50‐fold higher in plants expressing codon‐optimized VP1 (Figure 2). Our *in vivo* study also showed that vaccination with codon‐optimized VP1 induced significantly higher IgG1 and IgA antibody responses than the native VP1 gene, indicating that the higher antigen doses increase efficacy of oral immunization. Because we gave 20 mg of lyophilized plant cells for mice weighing 20–25 g, it is easy to deliver adequate dose to children. In this context, our recent success in producing antigens in a commercial cGMP facility should help in advancing this concept to the clinic (Su *et al*., 2015).

### Plant‐derived adjuvants and antigens

*Quillaia* extract (Type 1) contains over 100 plant‐derived triterpenoid saponins (20–26%) and has been approved as Substances Generally Recognized as Safe (GRAS) by FDA (Agency Response Letter GRAS Notice No. GRN 000165). Squalene is a substance naturally found in the human body, as well as in animals and plants and has been used as an adjuvant in FDA‐licensed AS03‐adjuvanted Influenza A (H5N1) virus monovalent vaccine (GlaxoSmithKline) and MF59‐adjuvanted influenza vaccine (Fluad^™^) (Novartis). Therefore, antigens and adjuvants used in this study have been previously or currently used in the clinic. Recombinant CTB is approved as a component of an internationally licensed oral cholera vaccine Dukoral^®^ and is used in the clinic for a decade (Hill *et al*., 2006). Furthermore, oral administration of rCTB significantly decreased inflammation in mild to moderately active Crohn's disease in human clinical trials (Stål *et al*., 2010). Oral delivery of rCTB chemically cross‐linked to a peptide from the human 60‐kD heat shock protein mitigated uveitis of Behcet's disease in a Phase I/II clinical trials (Stanford *et al*., 2004).

Modelling of VP1 3‐D structure shows that the key epitopes appear on the protein surface, demonstrating the suitability of this structure to generate antibodies suitable for neutralization (Liu *et al*., 2011). Neutralizing antibody levels at a titre above the 1 : 8 dilution [3 log_2_(titre)] threshold are accepted by all national regulatory agencies as having a good correlation with protection when reviewing licence applications for IPV‐containing vaccines (Plotkin, 2010; Verdijk *et al*., 2014). As expected, virus‐neutralizing titres induced by two IPV doses were high for all Sabin strains. In our study, priming with IPV and orally boosting with bioencapsulated VP1 with plant‐derived adjuvants (saponin and squalene) showed the highest seropositivity and virus‐neutralizing titres (range 3.17–10.17 log_2_ titre) against all Sabin 1, 2, 3 strains. Although mice that were only boosted with VP1 plus two adjuvants but not primed (group 10) showed the strong VP1‐specific antibody (IgG1 and IgA) production, no neutralizing virus titres were observed in this group when compared to the mice that were primed with IPV. Thus, only oral boosting with subunit vaccination appears to be insufficient to induce a good neutralizing antibody response to the antigen. Compared to VP1‐specific antibody (IgG1 and IgA), CTB‐specific IgG1 and IgA titres were negligible because mice were not primed with CTB. These results demonstrate that oral priming is essential to induce adequate immunity against pathogens. Unfortunately, most articles on the plant‐made vaccine literature report antibody titres but not results on pathogen or toxin challenge, leading to misleading conclusions on the requirement of priming (Chan and Daniell, 2015; Peréz Aguirreburualde *et al*., 2013).

### Need for other booster vaccines for the elderly population

Although this study focuses on polio booster vaccine, there is a greater need to boost immunity as life expectancy is on the rise. Loss of immunity against infectious diseases among elderly population is a growing concern. For example, shingles occur when latent chickenpox virus is reactivated when ageing weakens the immune system and this is rarely observed due to new viral infections. Therefore, to enhance immunity against a number of infectious diseases among elderly population, low‐cost oral booster vaccines could serve as a novel concept.

In conclusion, virus and cold chain‐free vaccines are not currently available for any infectious disease. Therefore, production and oral delivery of vaccines using transplastomic technology should facilitate the development of low‐cost cold chain‐ and virus‐free booster vaccines. Here, we show a low‐cost booster vaccine using bioencapsulated polio antigens as an alternative strategy to avoid repeated OPV vaccinations for global poliovirus eradication and prevention of polio outbreaks in endemic areas.

## Experimental procedures

### VP1 chloroplast vector construction and regeneration of transplastomic plants

The native VP1 gene (906 bp) of Sabin 1 poliovirus (Genbank accession number AY184219) was amplified using forward primer 5'‐gggCCCgggCCCCggCgTAAACgCTCTgTTgggTTAggTCAgATg‐3' and reverse primer 5'‐CgATCTAgATCAATATgTggTCAgATC‐3'. The PCR‐amplified fragment and the codon‐optimized VP1 gene (synthesized by GenScript, Piscataway, NJ) were cloned into tobacco and lettuce chloroplast transformation vectors. Biolistic delivery of chloroplast transformation vectors and regeneration of transplastomic tobacco (*Nicotiana tabacum* cv. Petit Havana) lines were performed as previously described (Verma *et al*., 2008).

### Characterization of transplastomic tobacco and lettuce lines

To confirm transgene cassette integration into the tobacco chloroplast genome, PCR was performed using primer pairs 3P/3M and 5P/2M (Verma *et al*., 2008). Southern blot analysis was performed to confirm transgene integration and homoplasmy as previously described (Verma *et al*., 2008).

### Lyophilization of CTB‐VP1 transplastomic tobacco leaves

Frozen CTB‐VP1 tobacco leaves were transported to a lyophilizer (Genesis 35XL, SP Scientific, Stone Ridge, NY) on dry ice and lyophilized at −40, −30, −20, −15, −10, −5 and 25 °C for a total of 72 h under a 400 mTorr vacuum. Lyophilized leaf materials were ground in a coffee grinder (Hamilton Beach, Southern Pines, NC) three times at maximum speed (pulse on 10 s and off 30 s). The fine powder was stored with silica gel in a moisture‐free environment at room temperature.

### Immunoblot analysis and purification of chloroplast‐derived proteins

Immunoblot analysis and quantitation of CTB‐VP1 fusion proteins were performed according to previously published methods (Davoodi‐Semiromi *et al*., 2010). The densitometry assay for quantification of CTB‐VP1 was carried out using known amount monomeric form (11.5 kDa) CTB standard (Sigma, St Louis, MO) and the monomer of the fusion protein. To detect CTB‐VP1‐fused proteins, blots were incubated with 1 : 10 000 rabbit anti‐CTB polyclonal antibody (GeneWay, San Diego, CA) or 1 : 1000 rabbit anti‐VP1 polyclonal antibody (Alpha Diagnostic Intl. Inc., San Antonio, TX) followed by 1 : 4000 goat anti‐rabbit IgG‐HRP as secondary antibody (SouthernBiotech, Birmingham, AL). CTB and recombinant Sabin 1 VP1 (Alpha Diagnostic Intl. Inc.) were used as positive controls. To purify chloroplast‐derived CTB‐VP1 fusion proteins, His60 Ni Superflow Resin (Clontech Laboratories, Mountain View, CA) was used according to the manufacturer's instructions. Eluted fractions were dialysed three times with sterile PBS, aliquoted and stored at −20 °C. Purified chloroplast‐derived CTB‐VP1 was used for immunoglobulin measurements.

### Cholera toxin B‐GM1‐ganglioside receptor binding assay

To test the ability of the tobacco chloroplast‐derived CTB‐VP1 to form pentamers and bind to the GM1‐ganglioside receptor, a CTB–GM1 binding assay was performed as described (Davoodi‐Semiromi *et al*., 2010).

### Mice and immunization schedule

Female CD‐1 mice aged 6–7 weeks were purchased from Charles River Laboratories (Wilmington, MA) and housed in microisolator cages. Experiments were conducted in accordance with guidelines of the University of Pennsylvania Institutional Animal Care and Use Committee. Mice were randomly divided into nine groups of 10 mice per group. Group 1 was a control group in which mice were untreated. All mice from groups 2 through 8 were subcutaneously (s.c.) primed with 100 μL of IPV suspension of three types of poliovirus [40 : 8 : 32 D antigen unit of type 1 (Mahoney), type 2 (MEF‐1) and type 3 (Saukett) (IPOL, Sanofi Pasteur, Swiftwater, PA)]. Group 2 mice were s.c. boosted with the same IPV 30 days after priming. Group 3 mice were only s.c. primed with IPV. Mice in groups 4 through 10 were orally boosted with lyophilized plant material: mice in groups 4–9 were boosted once a week for eight consecutive weeks starting 1 week after priming. Mice in groups 4 through 6 were orally boosted with lyophilized native CTB‐VP1‐expressing tobacco leaves; each mouse was boosted with 20 mg of material in 200 μL of PBS plus different adjuvants: saponin (Saponin from *quilaja bark*, Sigma) (group 4), squalene (Sigma) (group 5) or both (group 6). Mice in groups 7 through 10 were orally boosted with lyophilized codon‐optimized CTB‐VP1‐expressing tobacco leaves; each mouse was boosted with 20 mg of material in 200 μL of PBS plus different adjuvants: saponin (group 6), squalene (group 7) or both (groups 9 and 10). Blood was collected 1 day prior to priming and 10 days after boosting. Serum samples were heat‐inactivated at 56 °C for 30 min to destroy complement activity. In the third month after oral boosting with CTB‐VP1 formulations, faecal pellets were collected at day 10 after vaccination.

### Preparation of vaccine formulations of bioencapsulated, plant‐made CTB‐VP1

Vaccine formulation was generally performed as previously described (Domingos *et al*., 2008; Lee *et al*., 2015) but with modifications. Primary emulsion in the aqueous phase was made by mixing 0.05% Tween‐80 in PBS with 20 mg of lyophilized VP1 tobacco plant material (with 2 mg saponin or not) and followed by mixture with the oil phase of squalene (80% v/v squalene, 20% v/v Span‐80).

### Determination of antibody response by ELISA

Immunological responses, including serum and faecal extract levels of VP1‐specific IgG1, IgA and CTB‐specific IgG1, IgA titres, were assayed by direct ELISA as previously described (Davoodi‐Semiromi *et al*., 2010). Blood was collected 1 day prior to priming and 10 days after boosting. Faecal pellets were collected in the third month after oral boosting. As described previously (Kraynyak *et al*., 2010), all faecal pellets were weighed and 2 mL/g of 0.01% BSA, 0.02% sodium azide solution containing protease inhibitor was added. After vigorous vortexing (~20 min), samples were centrifuged at 135 ***g*** for 20 min. The supernatant was transferred to a clean tube for further centrifugation (1600 ***g***, 30 min). The supernatant was aliquoted and stored at −80 °C. Briefly, for measuring VP1‐IgG1 and VP1‐IgA antibody titres, 10 μg/mL purified CTB‐VP1 proteins was used for coating 96‐well Maxisorp ELISA plate (Nunc) overnight at 4 °C. For measuring CTB‐ IgG1 and CTB‐IgA antibody titres, commercial source cholera toxin B subunit (1 μg/mL) (Sigma) was used for coating 96‐well ELISA plate overnight at 4 °C. Plates were blocked with 1% BSA (Sigma 7906) in PBS with 0.05% Tween. Twofold dilution of heat‐inactivated individual mice sera samples (start at 1 : 400 dilution for VP1‐IgG1, 1 : 50 dilution for VP1‐IgA and CTB‐IgG1) or faecal pellet extracts (start at 1 : 5 for both VP1‐IgA and CTB‐IgA) were incubated overnight at 4 °C, followed by HRP‐conjugated rat anti‐mouse IgG1 (1 : 1000) (BD) or HRP‐conjugated goat anti‐mouse IgA (1 : 5000) (American Qualex, San Clemente, CA) diluted in blocking buffer and incubated at 37 °C for 1 h followed by colour development with TMB substrate for 10 min at RT. The reaction was stopped by adding 100 μL of 2N sulphuric acid per well, and absorbance was measured using an ELISA reader at 450 nm. Antibody titres are defined as the reciprocal of the highest dilution above the cut‐off, which was three times the mean background (Frey *et al*., 1998). All sera samples and faecal pellet extracts were tested in triplicate. Results are shown as individual antibody titre ± SEM.

### Poliovirus Sabin 1, 2, 3 neutralization assay

Animals were orally boosted with native or codon‐optimized CTB‐VP1 proteins adjuvanted with saponin and/or squalene or, for group 2 and group 3, both priming and boosting with the IPV or priming with IPV only. Ten days after the last oral boost (day 117 of the experiment), serum samples were collected and saved at −80 °C for further neutralization assays at Centers for Disease Control and Prevention (CDC) as previously described (Verdijk *et al*., 2014; WHO, 1991, 1997). Briefly, sera samples were tested in triplicate with the use of modified microneutralization assays for antibodies to Sabin strains type 1, 2 and 3. Serum samples from control and experimental groups were tested randomly and blindly. The serum dilution of a reciprocal titre at which no virus neutralization was detected was recorded as the log_2_ (titre) of 2.5, or negative; a log_2_ titre of ≥3 was considered protective. Individual titres for each mouse are plotted, and the bar represents mean neutralizing titre ± SEM.

### Statistical analysis

All data are reported for individual mice, and mean ± SEM is given for each group. Analyses for statistically significant differences in antibody titres between groups were performed using One‐way ANOVA and *t*‐test (GraphPad Prism version 6) and *P* values <0.05 were considered significant.

## Conflict of interest

Although there is no financial conflict of interest to report, it is disclosed that the corresponding author is an inventor on numerous patents reporting expression of human therapeutic proteins in chloroplasts.
